# *N*′-(4-Hy­droxy­benzyl­idene)ferrocene-1-carbohydrazide

**DOI:** 10.1107/S1600536811050835

**Published:** 2011-12-03

**Authors:** Wen-juan Li, Manman Song, Yan Xu

**Affiliations:** a Department of Chemistry, Zhengzhou University, Zhengzhou 450052, People’s Republic of China

## Abstract

In the title compound, [Fe(C_5_H_5_)_2_(C_13_H_11_N_2_O_2_)], the dihedral angle between the benzene ring and the cyclo­penta­diene ring bonded to the carbonyl group is 26.1 (2)°. In the crystal, bifurcated O—H⋯(O,N) and N—H⋯O hydrogen bonds link the mol­ecules into a three-dimensional network.

## Related literature

For background to ferrocenyl­carbonyl­hydrazone complexes and the synthesis of the title compound, see: Ma *et al.* (1988 ▶).
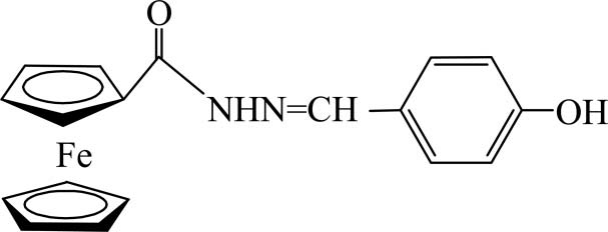


## Experimental

### 

#### Crystal data

[Fe(C_5_H_5_)_2_(C_13_H_11_N_2_O_2_)]
*M* *_r_* = 348.18
Orthorhombic, 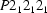

*a* = 11.341 (2) Å
*b* = 11.669 (2) Å
*c* = 11.748 (2) Å
*V* = 1554.7 (5) Å^3^
*Z* = 4
Mo *K*α radiation
μ = 0.98 mm^−1^
*T* = 293 K
0.21 × 0.18 × 0.17 mm

#### Data collection

Rigaku Saturn diffractometer
Absorption correction: multi-scan (*CrystalClear*; Rigaku/MSC, 2006 ▶) *T*_min_ = 0.821, *T*_max_ = 0.851
13023 measured reflections
3691 independent reflections
3139 reflections with *I* > 2σ(*I*)
*R*_int_ = 0.043

#### Refinement

*R*[*F* ^2^ > 2σ(*F* ^2^)] = 0.047
*wR*(*F* ^2^) = 0.097
*S* = 1.06
3691 reflections
208 parameters
H-atom parameters constrained
Δρ_max_ = 0.20 e Å^−3^
Δρ_min_ = −0.28 e Å^−3^
Absolute structure: Flack (1983 ▶), 1583 Friedel pairs
Flack parameter: 0.07 (2)

### 

Data collection: *CrystalClear* (Rigaku/MSC, 2006 ▶); cell refinement: *CrystalClear*; data reduction: *CrystalClear*; program(s) used to solve structure: *SHELXS97* (Sheldrick, 2008 ▶); program(s) used to refine structure: *SHELXL97* (Sheldrick, 2008 ▶); molecular graphics: *PLATON* (Spek, 2009 ▶); software used to prepare material for publication: *CrystalStructure* (Rigaku/MSC, 2006 ▶).

## Supplementary Material

Crystal structure: contains datablock(s) global, I. DOI: 10.1107/S1600536811050835/lh5370sup1.cif

Structure factors: contains datablock(s) I. DOI: 10.1107/S1600536811050835/lh5370Isup2.hkl

Additional supplementary materials:  crystallographic information; 3D view; checkCIF report

## Figures and Tables

**Table 1:** Hydrogen-bond geometry (Å, °)

*D*—H⋯*A*	*D*—H	H⋯*A*	*D*⋯*A*	*D*—H⋯*A*
N2—H2*B*⋯O1^i^	0.86	2.20	3.035 (3)	163
O1—H1*A*⋯O2^ii^	0.82	2.03	2.838 (3)	170
O1—H1*A*⋯N1^ii^	0.82	2.59	3.028 (3)	115

Symmetry codes: (i) 
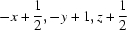
; (ii) 
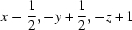
.

**Fig. 1.**
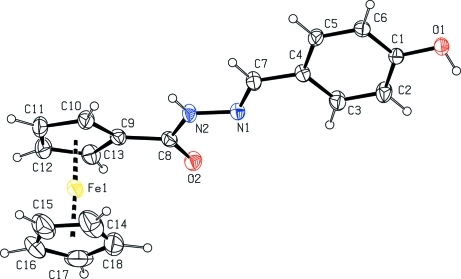
The molecular structure of the title compound with 30% displacement ellipsoids for non-H atoms.

## supplementary crystallographic information

### Comment

It is known that ferrocenyl-bearing hydrazones can form stable complexes with various transition metal ions (Ma *et al.*, 1988). To further explore these types of structures, we synthesized the title compound and its crystal structure is presented herein.

The molecular structure of the title compound is shown in Fig. 1. The distance between the two cyclopentadiene rings of the ferrocene is 3.2871 (4) Å. The distance between Fe1 and the mean-planes of the five-membered rings are 1.6377 (5) Å and 1.6498 (5) Å. The dihedral angle between the benzene ring and the cyclopentadiene ring bonded to the carbonyl group is 26.1 (2)°. In the crystal, bifurcated O—H···(O,N) and N—H···O hydrogen bonds link molecules into a three-dimensional network (Table 1).

### Experimental

The synthesis of the title compound followed the procedure of Ma *et al.* (1988). The title compound (0.02 mmol) was dissolved in acetonitrile (3 mL) with a little methanol. Slow evaportation at room temperature for two weeks gave red crystals.

### Refinement

All H atoms were placed in calculated positions, with C—H = 0.93-0.98 Å, N—H = 0.86Å, O—H = 0.82Å and included in the refinement in a riding-model approximation with *U*_iso_ = 1.2*U*_eq_(C,N) or 1.5*U*_eq_(O).

### Crystal data

**Table:**

[Fe(C_5_H_5_)_2_(C_13_H_11_N_2_O_2_)]	*F*(000) = 720
*M**_r_* = 348.18	*D*_x_ = 1.488 Mg m^−^^3^
Orthorhombic, *P*2_1_2_1_2_1_	Mo *K*α radiation, λ = 0.71073 Å
Hall symbol: P 2ac 2ab	Cell parameters from 4569 reflections
*a* = 11.341 (2) Å	θ = 2.5–2.5°
*b* = 11.669 (2) Å	µ = 0.98 mm^−^^1^
*c* = 11.748 (2) Å	*T* = 293 K
*V* = 1554.7 (5) Å^3^	Prism, red
*Z* = 4	0.21 × 0.18 × 0.17 mm

### Data collection

**Table:**

Rigaku Saturn diffractometer	3691 independent reflections
Radiation source: fine-focus sealed tube	3139 reflections with *I* > 2σ(*I*)
graphite	*R*_int_ = 0.043
Detector resolution: 28.5714 pixels mm^-1^	θ_max_ = 27.9°, θ_min_ = 2.5°
ω scans	*h* = −14→11
Absorption correction: multi-scan (*CrystalClear*; Rigaku/MSC, 2006)	*k* = −14→14
*T*_min_ = 0.821, *T*_max_ = 0.851	*l* = −15→15
13023 measured reflections	

### Refinement

**Table:**

Refinement on *F*^2^	Secondary atom site location: difference Fourier map
Least-squares matrix: full	Hydrogen site location: inferred from neighbouring sites
*R*[*F*^2^ > 2σ(*F*^2^)] = 0.047	H-atom parameters constrained
*wR*(*F*^2^) = 0.097	*w* = 1/[σ^2^(*F*_o_^2^) + (0.0432*P*)^2^] where *P* = (*F*_o_^2^ + 2*F*_c_^2^)/3
*S* = 1.06	(Δ/σ)_max_ < 0.001
3691 reflections	Δρ_max_ = 0.20 e Å^−^^3^
208 parameters	Δρ_min_ = −0.28 e Å^−^^3^
0 restraints	Absolute structure: Flack (1983), 1583 Friedel pairs
Primary atom site location: structure-invariant direct methods	Flack parameter: 0.07 (2)

### Special details

**Table:**

Geometry. All e.s.d.'s (except the e.s.d. in the dihedral angle between two l.s. planes) are estimated using the full covariance matrix. The cell e.s.d.'s are taken into account individually in the estimation of e.s.d.'s in distances, angles and torsion angles; correlations between e.s.d.'s in cell parameters are only used when they are defined by crystal symmetry. An approximate (isotropic) treatment of cell e.s.d.'s is used for estimating e.s.d.'s involving l.s. planes.
Refinement. Refinement of *F*^2^ against ALL reflections. The weighted *R*-factor *wR* and goodness of fit *S* are based on *F*^2^, conventional *R*-factors *R* are based on *F*, with *F* set to zero for negative *F*^2^. The threshold expression of *F*^2^ > σ(*F*^2^) is used only for calculating *R*-factors(gt) *etc*. and is not relevant to the choice of reflections for refinement. *R*-factors based on *F*^2^ are statistically about twice as large as those based on *F*, and *R*- factors based on ALL data will be even larger.

### Fractional atomic coordinates and isotropic or equivalent isotropic displacement parameters (Å^2^)

**Table:**

	*x*	*y*	*z*	*U*_iso_*/*U*_eq_	
C1	0.1100 (2)	0.3799 (2)	0.4653 (2)	0.0338 (6)	
C2	0.2016 (3)	0.3023 (3)	0.4696 (3)	0.0515 (9)	
H2A	0.2036	0.2415	0.4184	0.062*	
C3	0.2907 (3)	0.3143 (3)	0.5494 (3)	0.0508 (9)	
H3A	0.3513	0.2607	0.5521	0.061*	
C4	0.2904 (2)	0.4054 (3)	0.6253 (2)	0.0384 (6)	
C5	0.1976 (3)	0.4829 (3)	0.6188 (3)	0.0427 (8)	
H5A	0.1956	0.5447	0.6688	0.051*	
C6	0.1083 (3)	0.4706 (2)	0.5400 (3)	0.0424 (7)	
H6A	0.0471	0.5236	0.5374	0.051*	
C7	0.3819 (3)	0.4244 (2)	0.7082 (3)	0.0435 (7)	
H7A	0.3778	0.4906	0.7521	0.052*	
C8	0.6301 (3)	0.3160 (2)	0.8454 (2)	0.0374 (6)	
C9	0.7054 (2)	0.3576 (3)	0.9383 (3)	0.0381 (7)	
C10	0.7210 (3)	0.4729 (3)	0.9792 (3)	0.0479 (8)	
H10A	0.6798	0.5412	0.9510	0.058*	
C11	0.8062 (3)	0.4703 (3)	1.0672 (3)	0.0575 (9)	
H11A	0.8350	0.5367	1.1100	0.069*	
C12	0.8441 (3)	0.3547 (3)	1.0817 (3)	0.0573 (9)	
H12A	0.9036	0.3277	1.1360	0.069*	
C13	0.7833 (3)	0.2868 (3)	1.0025 (3)	0.0506 (8)	
H13A	0.7932	0.2040	0.9925	0.061*	
C14	0.9200 (4)	0.5014 (5)	0.7790 (5)	0.0972 (17)	
H14A	0.8701	0.5578	0.7402	0.117*	
C15	0.9991 (4)	0.5243 (5)	0.8663 (5)	0.0931 (16)	
H15A	1.0128	0.5998	0.9004	0.112*	
C16	1.0543 (3)	0.4238 (5)	0.8989 (4)	0.0871 (14)	
H16A	1.1137	0.4158	0.9590	0.105*	
C17	1.0091 (4)	0.3347 (5)	0.8301 (5)	0.0936 (16)	
H17A	1.0320	0.2538	0.8331	0.112*	
C18	0.9254 (4)	0.3842 (6)	0.7547 (4)	0.0965 (17)	
H18A	0.8803	0.3437	0.6960	0.116*	
Fe1	0.87527 (4)	0.40949 (4)	0.91860 (4)	0.04623 (14)	
N1	0.4684 (2)	0.3563 (2)	0.7252 (2)	0.0396 (6)	
N2	0.5467 (2)	0.3903 (2)	0.8087 (2)	0.0397 (6)	
H2B	0.5425	0.4581	0.8371	0.048*	
O1	0.02012 (17)	0.37099 (16)	0.38868 (17)	0.0424 (5)	
H1A	0.0456	0.3451	0.3286	0.064*	
O2	0.6370 (2)	0.21835 (16)	0.80566 (19)	0.0493 (5)	

### Atomic displacement parameters (Å^2^)

**Table:**

	*U*^11^	*U*^22^	*U*^33^	*U*^12^	*U*^13^	*U*^23^
C1	0.0353 (15)	0.0315 (14)	0.0345 (13)	−0.0021 (12)	−0.0008 (12)	0.0008 (11)
C2	0.056 (2)	0.0384 (17)	0.060 (2)	0.0098 (15)	−0.0180 (18)	−0.0153 (15)
C3	0.0468 (18)	0.0448 (18)	0.061 (2)	0.0154 (15)	−0.0174 (17)	−0.0116 (16)
C4	0.0348 (15)	0.0375 (15)	0.0430 (15)	−0.0012 (14)	−0.0057 (12)	−0.0004 (14)
C5	0.0427 (18)	0.0408 (17)	0.0446 (18)	0.0070 (14)	−0.0067 (14)	−0.0138 (14)
C6	0.0361 (17)	0.0418 (16)	0.0491 (17)	0.0104 (14)	−0.0057 (14)	−0.0099 (14)
C7	0.0413 (16)	0.0415 (15)	0.0478 (16)	−0.0003 (16)	−0.0109 (16)	−0.0044 (13)
C8	0.0307 (14)	0.0428 (15)	0.0386 (15)	−0.0030 (15)	0.0011 (13)	0.0010 (12)
C9	0.0335 (14)	0.0421 (16)	0.0388 (16)	0.0027 (13)	−0.0023 (13)	0.0018 (13)
C10	0.0463 (19)	0.0475 (19)	0.0500 (19)	0.0101 (15)	−0.0107 (16)	−0.0091 (16)
C11	0.051 (2)	0.065 (2)	0.057 (2)	0.0126 (17)	−0.0208 (18)	−0.0205 (19)
C12	0.055 (2)	0.073 (2)	0.0444 (17)	0.0098 (18)	−0.0180 (17)	−0.0001 (18)
C13	0.0500 (18)	0.053 (2)	0.049 (2)	0.0102 (16)	−0.0089 (16)	0.0089 (16)
C14	0.069 (3)	0.142 (5)	0.081 (3)	−0.026 (3)	−0.006 (3)	0.041 (3)
C15	0.059 (3)	0.112 (4)	0.108 (4)	−0.031 (3)	0.000 (3)	0.007 (3)
C16	0.037 (2)	0.132 (4)	0.092 (3)	−0.004 (3)	−0.008 (2)	−0.009 (3)
C17	0.068 (3)	0.109 (4)	0.103 (4)	0.013 (3)	0.036 (3)	−0.020 (3)
C18	0.067 (3)	0.166 (5)	0.056 (3)	−0.047 (3)	0.011 (2)	−0.018 (3)
Fe1	0.0355 (2)	0.0557 (3)	0.0474 (2)	−0.0009 (2)	−0.0079 (2)	0.0001 (2)
N1	0.0354 (13)	0.0474 (14)	0.0358 (13)	−0.0021 (12)	−0.0047 (11)	−0.0023 (11)
N2	0.0385 (13)	0.0375 (13)	0.0432 (14)	0.0065 (11)	−0.0143 (11)	−0.0045 (11)
O1	0.0376 (11)	0.0454 (12)	0.0441 (12)	0.0019 (9)	−0.0083 (9)	−0.0090 (9)
O2	0.0495 (12)	0.0403 (11)	0.0582 (14)	0.0066 (11)	−0.0090 (12)	−0.0118 (10)

### Geometric parameters (Å, °)

**Table:**

C1—O1	1.364 (3)	C11—H11A	0.9800
C1—C2	1.379 (4)	C12—C13	1.403 (5)
C1—C6	1.375 (4)	C12—Fe1	2.051 (4)
C2—C3	1.385 (4)	C12—H12A	0.9800
C2—H2A	0.9300	C13—Fe1	2.027 (3)
C3—C4	1.388 (4)	C13—H13A	0.9800
C3—H3A	0.9300	C14—C18	1.399 (7)
C4—C5	1.390 (4)	C14—C15	1.389 (7)
C4—C7	1.440 (4)	C14—Fe1	2.025 (5)
C5—C6	1.379 (4)	C14—H14A	0.9800
C5—H5A	0.9300	C15—C16	1.384 (7)
C6—H6A	0.9300	C15—Fe1	2.036 (4)
C7—N1	1.279 (4)	C15—H15A	0.9800
C7—H7A	0.9300	C16—C17	1.413 (7)
C8—O2	1.233 (3)	C16—Fe1	2.050 (4)
C8—N2	1.354 (4)	C16—H16A	0.9800
C8—C9	1.469 (4)	C17—C18	1.420 (7)
C9—C13	1.425 (4)	C17—Fe1	2.037 (4)
C9—C10	1.440 (4)	C17—H17A	0.9800
C9—Fe1	2.032 (3)	C18—Fe1	2.029 (4)
C10—C11	1.416 (4)	C18—H18A	0.9800
C10—Fe1	2.029 (3)	N1—N2	1.381 (3)
C10—H10A	0.9800	N2—H2B	0.8600
C11—C12	1.426 (5)	O1—H1A	0.8200
C11—Fe1	2.041 (4)		
			
O1—C1—C2	122.4 (2)	C15—C16—Fe1	69.7 (2)
O1—C1—C6	118.0 (2)	C17—C16—Fe1	69.2 (2)
C2—C1—C6	119.5 (3)	C15—C16—H16A	126.2
C1—C2—C3	120.5 (3)	C17—C16—H16A	126.2
C1—C2—H2A	119.8	Fe1—C16—H16A	126.2
C3—C2—H2A	119.8	C16—C17—C18	107.5 (5)
C4—C3—C2	120.7 (3)	C16—C17—Fe1	70.3 (2)
C4—C3—H3A	119.6	C18—C17—Fe1	69.3 (3)
C2—C3—H3A	119.6	C16—C17—H17A	126.3
C3—C4—C5	117.7 (3)	C18—C17—H17A	126.3
C3—C4—C7	123.4 (3)	Fe1—C17—H17A	126.3
C5—C4—C7	118.8 (3)	C17—C18—C14	107.5 (4)
C6—C5—C4	121.7 (3)	C17—C18—Fe1	69.8 (3)
C6—C5—H5A	119.2	C14—C18—Fe1	69.6 (3)
C4—C5—H5A	119.2	C17—C18—H18A	126.3
C1—C6—C5	119.9 (3)	C14—C18—H18A	126.3
C1—C6—H6A	120.1	Fe1—C18—H18A	126.3
C5—C6—H6A	120.1	C14—Fe1—C13	153.77 (19)
N1—C7—C4	124.3 (3)	C14—Fe1—C9	119.18 (17)
N1—C7—H7A	117.9	C13—Fe1—C9	41.10 (12)
C4—C7—H7A	117.9	C14—Fe1—C10	107.86 (19)
O2—C8—N2	121.1 (3)	C13—Fe1—C10	69.11 (13)
O2—C8—C9	123.3 (3)	C9—Fe1—C10	41.53 (12)
N2—C8—C9	115.5 (2)	C14—Fe1—C11	127.2 (2)
C13—C9—C10	106.8 (3)	C13—Fe1—C11	68.42 (15)
C13—C9—C8	124.2 (3)	C9—Fe1—C11	69.04 (13)
C10—C9—C8	128.9 (3)	C10—Fe1—C11	40.71 (12)
C13—C9—Fe1	69.24 (18)	C14—Fe1—C18	40.4 (2)
C10—C9—Fe1	69.11 (18)	C13—Fe1—C18	120.18 (19)
C8—C9—Fe1	124.4 (2)	C9—Fe1—C18	109.30 (15)
C9—C10—C11	107.9 (3)	C10—Fe1—C18	128.87 (19)
C9—C10—Fe1	69.36 (17)	C11—Fe1—C18	165.9 (2)
C11—C10—Fe1	70.1 (2)	C14—Fe1—C17	68.1 (2)
C9—C10—H10A	126.1	C13—Fe1—C17	109.22 (18)
C11—C10—H10A	126.1	C9—Fe1—C17	129.56 (19)
Fe1—C10—H10A	126.1	C10—Fe1—C17	168.0 (2)
C12—C11—C10	108.3 (3)	C11—Fe1—C17	150.7 (2)
C12—C11—Fe1	70.0 (2)	C18—Fe1—C17	40.9 (2)
C10—C11—Fe1	69.2 (2)	C14—Fe1—C16	67.5 (2)
C12—C11—H11A	125.8	C13—Fe1—C16	128.46 (18)
C10—C11—H11A	125.8	C9—Fe1—C16	167.32 (19)
Fe1—C11—H11A	125.8	C10—Fe1—C16	149.77 (18)
C13—C12—C11	107.8 (3)	C11—Fe1—C16	116.65 (17)
C13—C12—Fe1	68.9 (2)	C18—Fe1—C16	68.10 (18)
C11—C12—Fe1	69.2 (2)	C17—Fe1—C16	40.46 (19)
C13—C12—H12A	126.1	C14—Fe1—C15	40.01 (19)
C11—C12—H12A	126.1	C13—Fe1—C15	165.12 (19)
Fe1—C12—H12A	126.1	C9—Fe1—C15	152.17 (19)
C12—C13—C9	109.2 (3)	C10—Fe1—C15	117.4 (2)
C12—C13—Fe1	70.8 (2)	C11—Fe1—C15	107.1 (2)
C9—C13—Fe1	69.66 (18)	C18—Fe1—C15	67.4 (2)
C12—C13—H13A	125.4	C17—Fe1—C15	67.3 (2)
C9—C13—H13A	125.4	C16—Fe1—C15	39.6 (2)
Fe1—C13—H13A	125.4	C14—Fe1—C12	164.9 (2)
C18—C14—C15	108.0 (5)	C13—Fe1—C12	40.26 (13)
C18—C14—Fe1	70.0 (3)	C9—Fe1—C12	68.74 (13)
C15—C14—Fe1	70.4 (3)	C10—Fe1—C12	68.75 (13)
C18—C14—H14A	126.0	C11—Fe1—C12	40.80 (14)
C15—C14—H14A	126.0	C18—Fe1—C12	152.8 (2)
Fe1—C14—H14A	126.0	C17—Fe1—C12	118.2 (2)
C16—C15—C14	109.5 (5)	C16—Fe1—C12	107.55 (17)
C16—C15—Fe1	70.8 (3)	C15—Fe1—C12	127.30 (19)
C14—C15—Fe1	69.5 (3)	C7—N1—N2	115.2 (2)
C16—C15—H15A	125.2	C8—N2—N1	119.4 (2)
C14—C15—H15A	125.2	C8—N2—H2B	120.3
Fe1—C15—H15A	125.2	N1—N2—H2B	120.3
C15—C16—C17	107.5 (4)	C1—O1—H1A	109.5
			
O1—C1—C2—C3	−179.6 (3)	C11—C10—Fe1—C9	119.0 (3)
C6—C1—C2—C3	1.0 (5)	C9—C10—Fe1—C11	−119.0 (3)
C1—C2—C3—C4	−1.0 (6)	C9—C10—Fe1—C18	74.3 (3)
C2—C3—C4—C5	0.5 (5)	C11—C10—Fe1—C18	−166.7 (3)
C2—C3—C4—C7	−178.2 (3)	C9—C10—Fe1—C17	46.0 (10)
C3—C4—C5—C6	0.1 (5)	C11—C10—Fe1—C17	165.0 (9)
C7—C4—C5—C6	178.8 (3)	C9—C10—Fe1—C16	−170.1 (3)
O1—C1—C6—C5	−179.9 (3)	C11—C10—Fe1—C16	−51.1 (4)
C2—C1—C6—C5	−0.4 (5)	C9—C10—Fe1—C15	156.4 (2)
C4—C5—C6—C1	−0.1 (5)	C11—C10—Fe1—C15	−84.6 (3)
C3—C4—C7—N1	−4.5 (5)	C9—C10—Fe1—C12	−81.5 (2)
C5—C4—C7—N1	176.8 (3)	C11—C10—Fe1—C12	37.5 (2)
O2—C8—C9—C13	−8.5 (5)	C12—C11—Fe1—C14	−167.3 (2)
N2—C8—C9—C13	168.2 (3)	C10—C11—Fe1—C14	73.0 (3)
O2—C8—C9—C10	168.3 (3)	C12—C11—Fe1—C13	37.09 (19)
N2—C8—C9—C10	−15.0 (5)	C10—C11—Fe1—C13	−82.6 (2)
O2—C8—C9—Fe1	78.4 (3)	C12—C11—Fe1—C9	81.3 (2)
N2—C8—C9—Fe1	−104.9 (3)	C10—C11—Fe1—C9	−38.39 (19)
C13—C9—C10—C11	−0.6 (4)	C12—C11—Fe1—C10	119.7 (3)
C8—C9—C10—C11	−177.8 (3)	C12—C11—Fe1—C18	167.2 (6)
Fe1—C9—C10—C11	−59.8 (2)	C10—C11—Fe1—C18	47.5 (7)
C13—C9—C10—Fe1	59.2 (2)	C12—C11—Fe1—C17	−54.0 (4)
C8—C9—C10—Fe1	−118.0 (3)	C10—C11—Fe1—C17	−173.7 (4)
C9—C10—C11—C12	0.1 (4)	C12—C11—Fe1—C16	−86.3 (3)
Fe1—C10—C11—C12	−59.2 (3)	C10—C11—Fe1—C16	154.0 (2)
C9—C10—C11—Fe1	59.3 (2)	C12—C11—Fe1—C15	−127.9 (2)
C10—C11—C12—C13	0.5 (4)	C10—C11—Fe1—C15	112.4 (3)
Fe1—C11—C12—C13	−58.3 (3)	C10—C11—Fe1—C12	−119.7 (3)
C10—C11—C12—Fe1	58.8 (3)	C17—C18—Fe1—C14	118.5 (4)
C11—C12—C13—C9	−0.8 (4)	C17—C18—Fe1—C13	−84.8 (3)
Fe1—C12—C13—C9	−59.3 (2)	C14—C18—Fe1—C13	156.6 (3)
C11—C12—C13—Fe1	58.4 (3)	C17—C18—Fe1—C9	−128.8 (3)
C10—C9—C13—C12	0.9 (4)	C14—C18—Fe1—C9	112.7 (3)
C8—C9—C13—C12	178.2 (3)	C17—C18—Fe1—C10	−171.4 (3)
Fe1—C9—C13—C12	60.0 (3)	C14—C18—Fe1—C10	70.1 (3)
C10—C9—C13—Fe1	−59.1 (2)	C17—C18—Fe1—C11	150.5 (6)
C8—C9—C13—Fe1	118.2 (3)	C14—C18—Fe1—C11	32.0 (8)
C18—C14—C15—C16	−0.5 (6)	C14—C18—Fe1—C17	−118.5 (4)
Fe1—C14—C15—C16	59.7 (4)	C17—C18—Fe1—C16	38.0 (3)
C18—C14—C15—Fe1	−60.2 (3)	C14—C18—Fe1—C16	−80.6 (3)
C14—C15—C16—C17	0.2 (6)	C17—C18—Fe1—C15	80.9 (3)
Fe1—C15—C16—C17	59.1 (3)	C14—C18—Fe1—C15	−37.7 (3)
C14—C15—C16—Fe1	−58.9 (3)	C17—C18—Fe1—C12	−48.0 (5)
C15—C16—C17—C18	0.2 (5)	C14—C18—Fe1—C12	−166.5 (3)
Fe1—C16—C17—C18	59.6 (3)	C16—C17—Fe1—C14	80.6 (3)
C15—C16—C17—Fe1	−59.4 (3)	C18—C17—Fe1—C14	−37.8 (3)
C16—C17—C18—C14	−0.5 (5)	C16—C17—Fe1—C13	−127.3 (3)
Fe1—C17—C18—C14	59.7 (3)	C18—C17—Fe1—C13	114.3 (3)
C16—C17—C18—Fe1	−60.2 (3)	C16—C17—Fe1—C9	−169.0 (3)
C15—C14—C18—C17	0.6 (5)	C18—C17—Fe1—C9	72.6 (4)
Fe1—C14—C18—C17	−59.8 (3)	C16—C17—Fe1—C10	152.8 (8)
C15—C14—C18—Fe1	60.4 (3)	C18—C17—Fe1—C10	34.4 (11)
C18—C14—Fe1—C13	−50.9 (5)	C16—C17—Fe1—C11	−47.4 (5)
C15—C14—Fe1—C13	−169.5 (4)	C18—C17—Fe1—C11	−165.8 (4)
C18—C14—Fe1—C9	−85.8 (3)	C16—C17—Fe1—C18	118.4 (5)
C15—C14—Fe1—C9	155.5 (3)	C18—C17—Fe1—C16	−118.4 (5)
C18—C14—Fe1—C10	−129.7 (3)	C16—C17—Fe1—C15	37.2 (3)
C15—C14—Fe1—C10	111.7 (3)	C18—C17—Fe1—C15	−81.2 (3)
C18—C14—Fe1—C11	−170.7 (3)	C16—C17—Fe1—C12	−84.2 (3)
C15—C14—Fe1—C11	70.7 (4)	C18—C17—Fe1—C12	157.4 (3)
C15—C14—Fe1—C18	−118.6 (5)	C15—C16—Fe1—C14	36.8 (3)
C18—C14—Fe1—C17	38.3 (3)	C17—C16—Fe1—C14	−82.1 (4)
C15—C14—Fe1—C17	−80.3 (4)	C15—C16—Fe1—C13	−167.5 (3)
C18—C14—Fe1—C16	82.2 (3)	C17—C16—Fe1—C13	73.5 (4)
C15—C14—Fe1—C16	−36.5 (3)	C15—C16—Fe1—C9	161.2 (7)
C18—C14—Fe1—C15	118.6 (5)	C17—C16—Fe1—C9	42.2 (9)
C18—C14—Fe1—C12	155.8 (6)	C15—C16—Fe1—C10	−50.2 (5)
C15—C14—Fe1—C12	37.2 (8)	C17—C16—Fe1—C10	−169.2 (3)
C12—C13—Fe1—C14	−169.6 (4)	C15—C16—Fe1—C11	−84.8 (3)
C9—C13—Fe1—C14	−49.6 (5)	C17—C16—Fe1—C11	156.2 (3)
C12—C13—Fe1—C9	−120.0 (3)	C15—C16—Fe1—C18	80.6 (4)
C12—C13—Fe1—C10	−81.4 (2)	C17—C16—Fe1—C18	−38.3 (3)
C9—C13—Fe1—C10	38.61 (18)	C15—C16—Fe1—C17	118.9 (5)
C12—C13—Fe1—C11	−37.58 (19)	C17—C16—Fe1—C15	−118.9 (5)
C9—C13—Fe1—C11	82.4 (2)	C15—C16—Fe1—C12	−128.0 (3)
C12—C13—Fe1—C18	154.9 (3)	C17—C16—Fe1—C12	113.1 (3)
C9—C13—Fe1—C18	−85.1 (3)	C16—C15—Fe1—C14	−120.5 (5)
C12—C13—Fe1—C17	111.2 (3)	C16—C15—Fe1—C13	41.2 (9)
C9—C13—Fe1—C17	−128.8 (2)	C14—C15—Fe1—C13	161.7 (6)
C12—C13—Fe1—C16	70.0 (3)	C16—C15—Fe1—C9	−171.3 (3)
C9—C13—Fe1—C16	−170.0 (2)	C14—C15—Fe1—C9	−50.8 (6)
C12—C13—Fe1—C15	37.6 (8)	C16—C15—Fe1—C10	154.2 (3)
C9—C13—Fe1—C15	157.6 (7)	C14—C15—Fe1—C10	−85.4 (4)
C9—C13—Fe1—C12	120.0 (3)	C16—C15—Fe1—C11	111.4 (3)
C13—C9—Fe1—C14	157.3 (3)	C14—C15—Fe1—C11	−128.1 (3)
C10—C9—Fe1—C14	−84.2 (3)	C16—C15—Fe1—C18	−82.5 (3)
C8—C9—Fe1—C14	39.3 (3)	C14—C15—Fe1—C18	38.0 (3)
C10—C9—Fe1—C13	118.4 (3)	C16—C15—Fe1—C17	−38.0 (3)
C8—C9—Fe1—C13	−118.0 (3)	C14—C15—Fe1—C17	82.5 (4)
C13—C9—Fe1—C10	−118.4 (3)	C14—C15—Fe1—C16	120.5 (5)
C8—C9—Fe1—C10	123.6 (3)	C16—C15—Fe1—C12	70.9 (4)
C13—C9—Fe1—C11	−80.8 (2)	C14—C15—Fe1—C12	−168.6 (3)
C10—C9—Fe1—C11	37.65 (19)	C13—C12—Fe1—C14	162.1 (6)
C8—C9—Fe1—C11	161.2 (3)	C11—C12—Fe1—C14	42.3 (7)
C13—C9—Fe1—C18	114.1 (3)	C11—C12—Fe1—C13	−119.8 (3)
C10—C9—Fe1—C18	−127.4 (3)	C13—C12—Fe1—C9	37.65 (18)
C8—C9—Fe1—C18	−3.8 (3)	C11—C12—Fe1—C9	−82.1 (2)
C13—C9—Fe1—C17	72.7 (3)	C13—C12—Fe1—C10	82.4 (2)
C10—C9—Fe1—C17	−168.9 (3)	C11—C12—Fe1—C10	−37.42 (19)
C8—C9—Fe1—C17	−45.3 (4)	C13—C12—Fe1—C11	119.8 (3)
C13—C9—Fe1—C16	38.3 (8)	C13—C12—Fe1—C18	−53.4 (4)
C10—C9—Fe1—C16	156.7 (7)	C11—C12—Fe1—C18	−173.2 (3)
C8—C9—Fe1—C16	−79.7 (8)	C13—C12—Fe1—C17	−86.9 (3)
C13—C9—Fe1—C15	−167.9 (4)	C11—C12—Fe1—C17	153.3 (2)
C10—C9—Fe1—C15	−49.4 (4)	C13—C12—Fe1—C16	−129.5 (2)
C8—C9—Fe1—C15	74.1 (5)	C11—C12—Fe1—C16	110.7 (3)
C13—C9—Fe1—C12	−36.9 (2)	C13—C12—Fe1—C15	−168.7 (3)
C10—C9—Fe1—C12	81.5 (2)	C11—C12—Fe1—C15	71.6 (3)
C8—C9—Fe1—C12	−154.9 (3)	C4—C7—N1—N2	−180.0 (3)
C9—C10—Fe1—C14	114.1 (2)	O2—C8—N2—N1	−0.2 (4)
C11—C10—Fe1—C14	−126.9 (3)	C9—C8—N2—N1	−177.0 (2)
C9—C10—Fe1—C13	−38.22 (18)	C7—N1—N2—C8	169.6 (3)
C11—C10—Fe1—C13	80.8 (2)		

### Hydrogen-bond geometry (Å, °)

**Table:**

*D*—H···*A*	*D*—H	H···*A*	*D*···*A*	*D*—H···*A*
N2—H2B···O1^i^	0.86	2.20	3.035 (3)	163.
O1—H1A···O2^ii^	0.82	2.03	2.838 (3)	170.
O1—H1A···N1^ii^	0.82	2.59	3.028 (3)	115.

Symmetry codes: (i) −*x*+1/2, −*y*+1, *z*+1/2; (ii) *x*−1/2, −*y*+1/2, −*z*+1.
